# JAK/STAT pathway and molecular mechanism in bone remodeling

**DOI:** 10.1007/s11033-020-05910-9

**Published:** 2020-10-24

**Authors:** Eliana Rita Sanpaolo, Cinzia Rotondo, Daniela Cici, Ada Corrado, Francesco Paolo Cantatore

**Affiliations:** Department of Medical and Surgical Sciences, Rheumatology Clinic, University of Foggia Medical School, Foggia, Italy

**Keywords:** JAK/STAT pathway, Bone, Osteoblast, Osteoclast, Cytokine

## Abstract

JAK/STAT signaling pathway is involved in many diseases, including autoimmune diseases, which are characterized by a close interconnection between immune and bone system. JAK/STAT pathway is involved in bone homeostasis and plays an important role in proliferation and differentiation of some cell types, including osteoblasts and osteoclasts. Different molecules, such as cytokines, hormones, and growth factors are responsible for the activation of the JAK/STAT pathway, which leads, at the nuclear level, to start DNA transcription of target genes. Bone cells and remodeling process are often influenced by many cytokines, which act as strong stimulators of bone formation and resorption. Our aim, through careful research in literature, has been to provide an overview of the role of the JAK/STAT pathway in bone remodeling and on bone cells, with a focus on cytokines involved in bone turnover through this signal cascade. The JAK/STAT pathway, through the signal cascade activation mediated by the interaction with many cytokines, acts on bone cells and appears to be involved in bone remodeling process. However, many other studies are needed to completely understand the molecular mechanism underlying these bone process.

## Introduction

**Fig. 1:**
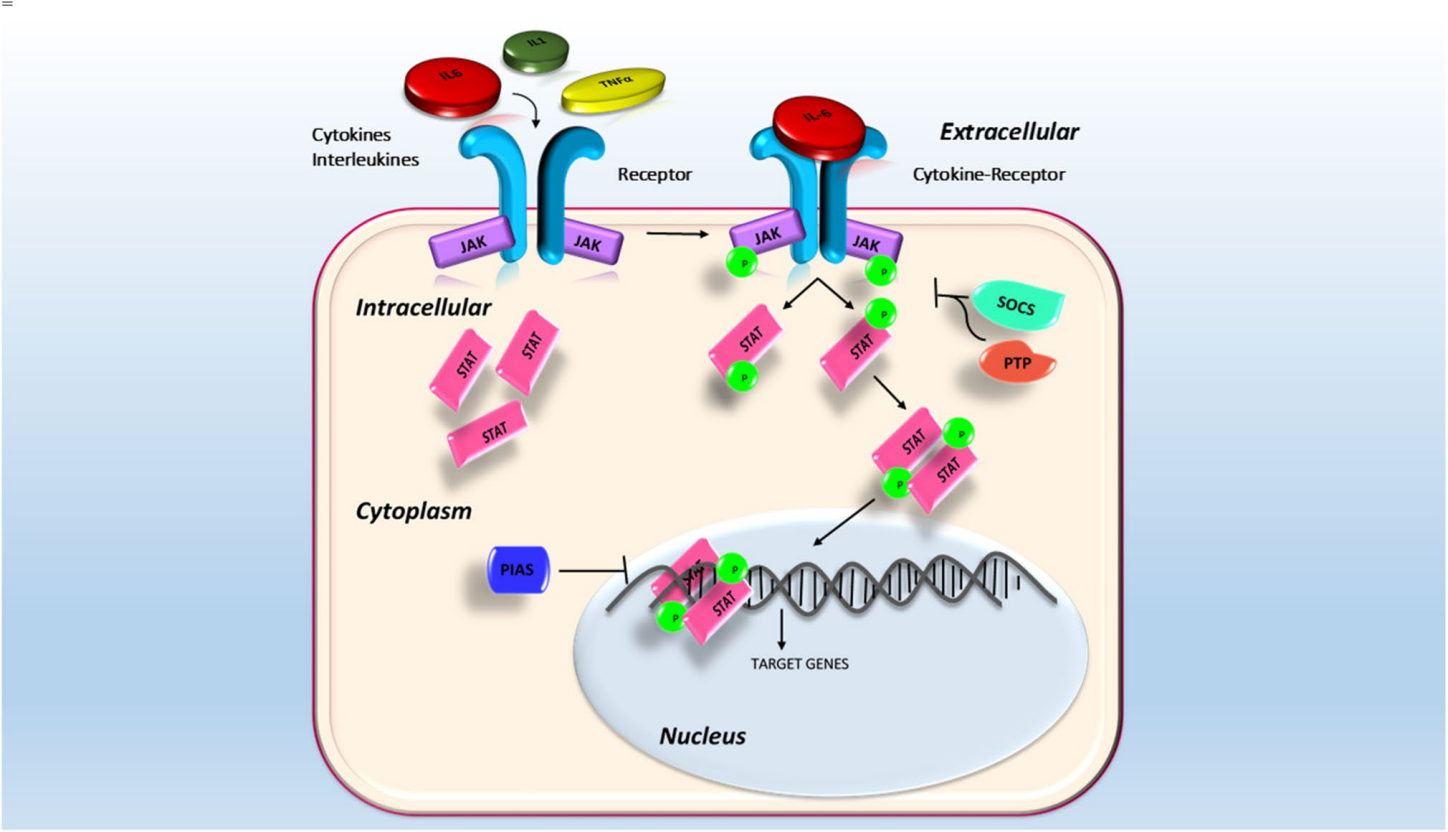
JAK/STAT pathway signaling. The binding of the cytokine to its receptor induces the activation of the JAKs, which phosphorylate the STATs elements. After the formation of STAT dimers, they migrate to the nucleus and, by binding to the target genes, they modify the transcription of DNA. Some elements can regulate the signaling cascade

The Janus kinases (JAKs) are a family of protein tyrosine kinases (PTKs), named JAK1, JAK2, JAK3, and TYK2, that act on signal transducer and activator of transcription (STAT). The expression of JAK3 appears to be mainly in the hematopoietic cells. In contrast, the expression of the other members, JAK1, JAK2, and TYK2, is ubiquitous. After their activation, JAKs induce the phosphorylation of some STAT elements in the cytoplasm, which, subsequently their dimerization, are translocated in the nucleus. In the nucleus, STAT dimers bind to specific areas of DNA leading to the regulation of target genes responsible for the regulation of migration, proliferation, and apoptosis [1–3]. JAK/STAT signaling pathway (Fig. 1) plays a key role in several cytokines, immune system regulators, hormones, and hematopoiesis factors [4]. A specific receptor is located on the surface of target cells that bind specific cytokines. These receptors, which can be composed of multiple subunits, are substantially associated with JAK monomer [5–9]. Initially, the JAK monomers appear to be inactive; consequently, the binding between the ligand and its receptor induces a JAK transphosphorylation receptor-associated and therefore its activation. Each member of the JAK family consists of four domains, including the SH2 domain, which is responsible for the link between the receptor and STAT member. In the cytoplasmic compartment, some receptor tyrosine residues are subject to an activated JAK-induced phosphorylation process. This process involves the formation of docking sites for the subsequent binding of the STAT components. The STAT family consists of seven members, named STAT1, STAT2, STAT3, STAT4, STAT5A, STAT5B, and STAT6, and each of these participates in the signal cascade depending on which cytokine binds to its receptor on the cell surface [10, 11]. Each STAT member is characterized by some domains, which perform specific functions in the activation and transcription process. N-terminal and SH2 domains are responsible for the binding and interactions between dimers and proteins. Besides N-terminal domain is involved in STAT phosphorylation. STATs link DNA of target genes through the DNA binding domain, forming a protein-DNA complex. Lastly, the C-terminal transcription domain contains highly conserved phosphorylated tyrosine (Y) and serine (S) residues required for STATs activation [12–15]. Through careful research in the literature, our purpose is to review how the JAK/STAT signaling pathway is involved in bone remodeling, how it acts at the cellular level, especially in osteoblasts and osteoclasts, and finally in which cytokines involved in the pathway may affect bone homeostasis.

## JAK/STAT negative factors

The activation of the JAK/STAT pathway is negatively regulated by a class of proteins called suppressor of cytokine signaling (SOCs), protein tyrosine phosphatase (PTP), and protein inhibitors of activated STAT (PIAS). SOCs protein class includes eight members named as follows: SOCS1, SOCS2, SOCS3, SOCS4, SOCS5, SOCS6, SOCS7 and CIS (cytokine-inducible SH2-containing protein) [16]. Each member of the family is made up of three common domains: a central SH domain, a C-terminal SOCS box, and a variable N-terminal domain. The conserved central domain SH2 is responsible for binding SOCs proteins with cytokine receptors. In this way, the activity of the JAK kinases is blocked and the whole signaling complex undergoes proteasome-mediated degradation. In addition, two members of the SOCS class, SOCS1, and SOCS3 contain a kinase inhibitory region (KIR), so, when they hook to the receptors, the catalytic activity of JAKs is inhibited [17–19]. The N-terminal domain is variable in the different members of SOCS, with different lengths in each of them. In fact, it has been seen that the N-terminal region of SOCS4-7 is longer than the other member of the family [20]. In the end, a highly conserved C-terminal SOCS box domain can enroll factors to the formation of ligase complex that leads to proteasomal degradation of ubiquitinylated proteins [21, 22].

PTP proteins belong to a large family of protein tyrosine phosphatase, whose function is mainly to cut out the phosphate group from tyrosine residues of phosphorylated proteins. PTPs have a catalytic domain within which a site-active sequence is positioned. Based on the differences in the aminoacidic sequence in the catalytic domain, they are divided into 4 classes (I, II, III, and IV) [23–26]. Protein inhibitors of activated STAT (PIAS), as the name says, act on STATs proteins by inhibiting their transcriptional activity. The members of the PIAS family are five: PIAS1, PIASxα, PIASxβ, PIAS3, and PIAS4 (PIASγ) [27, 28]. Some studies show that every protein can interact with a particular STAT member. For example, PIAS1 and PIAS4 are capable of interacting with STAT1, while PIAS3 with STAT3 and STAT5. When PIAS proteins connect to STAT members, they induce the blockade of the link between STATs and DNA. Thus, the transcription co-factors of the STAT target gene are not recruited. In this way, PIAS proteins negatively regulate the STATs transcriptional activity [29–32].

## JAK/STAT and bone cells

Many studies show that the JAK/STAT signaling pathway is involved in many diseases, including autoimmune diseases, and that its inhibition may be a therapeutic strategy. In autoimmune diseases, such as rheumatoid arthritis, the immune system, and the bone system are closely interconnected [33, 34]. The JAK/STAT pathway not only appears to be involved in bone homeostasis and response but also in the differentiation processes of some cells type. In particular, even the cytokines involved in the signaling pathway activation can act on this variety of cells, for example on osteoblasts, osteoclasts and osteocytes [35, 36]. The bone and the cells that regulate its remodeling are often subject to and/or influenced by the presence of cytokines belonging to the immune system. Many of them are strong modulators of bone metabolism, to influence the processes of bone formation and resorption. The bone physiological remodeling mechanism is due to a perfect relationship between osteoblasts and osteoclasts. These cells are responsible for bone formation and resorption mechanisms, respectively, while the role of mechanosensing is attributed to osteocytes [36]. Although both cells type cooperates in this process, they have different origins; while osteoblasts and osteocytes are of mesenchymal origin, osteoclasts, on the other hand, derive from hematopoietic stem cells [37, 38].

Osteoblasts are responsible for bone formation process, protein production, and matrix mineralization [39, 40]. During the differentiation process of osteoblasts, two transcriptional factors are very expressed. Shreds of evidence that these two factors, Runt-related transcription factor 2 (Runx2) and osterix (Osx), are necessary for the regulation of differentiation and are involved in maintaining of osteoblasts functionality [41, 42]. However, the role of osteoblasts also concerns the regulation of osteoclast differentiation. Indeed, when these two types of cells interact, osteoblasts produce several soluble factors, among which macrophage colony-stimulating factors (M-CSF), receptor activator of nuclear factor-κB ligand (RANKL), and osteoprotegerin (OPG) [43]. During the osteoclastogenesis process, RANKL is an essential factor, as it binds to the RANK receptor present on the surface of the precursors of the osteoclasts. This interaction also induces an increase in bone resorption capacity. In contrast, the RANK/RANKL interaction is negatively regulated by the presence of OPG. This molecule has a binding affinity with RANKL, thus, it prevents further binding (leads to a lower binding interaction) between the ligand and its receptor on osteoclasts [44, 45].

Recently, several studies have shown that different factors are involved during the osteoclastogenesis process [46]. This process leads to maturation and differentiation of osteoclasts from the monocyte/macrophage lineage. These cells are induced to differentiate starting from two factors, nuclear factor of activated T cells, cytoplasmic 1 (NFATc1) and c-Fos, whose expression is promoted by macrophage colony-stimulating factor (M-CSF) and receptor activator of nuclear factor-κB ligand (RANKL). The main osteoclasts signaling pathway provides a signal cascade that includes several factors including TNF receptor-associated factors (TRAFs), mitogen-activated protein kinases (MAPKs), and consequently the activation of c-Fos factor that induces the activity of NFATc1 [47]. The role of NFATc1 is considered significant for the promotion of differentiation and activity of osteoclasts [48–51]. Indeed, some studies have reported that downregulation of NFATc1 due to PIAS3 compromises the osteoclastogenesis process, confirming that this factor is necessary for the differentiation of active osteoclasts [52–54].

## Implication of JAK/STAT in bone metabolism

JAKs and STATs proteins, according to many studies, play an important role in the proliferation and differentiation of osteoblasts and osteoclasts. A murine study conducted on osteoblasts [55] has demonstrated that phosphorylation of JAKs proteins occurs in the presence of oncostatin-M. This indicates the involvement of the three members, JAK1, JAK2, and TYK2, in the bone formation process [1, 56–59]. Nevertheless, despite the expression of JAK3 is mainly limited to the leukocytes, its probable role in the bone remodeling process has recently been highlighted [37]. Other animal studies, conducted on JAK1-deficient mice, suggest that the absence of the JAK1 factor induces lower bone growth and significantly reduced body mass [60]. Experimental evidence shows that the role of the JAK2 member is determined by coupling with the STAT5B factor [61, 62]. A JAK2/STAT5B signaling pathway is very important in the mechanism of growth hormone (GH) signaling which, in turn, regulates osteoblasts differentiation. Furthermore, STAT5B is considered a transcriptional promoter of insulin-like growth factor 1 (IGF-1), which is also produced in osteoblasts and is involved as a mediator during the bone growth process [63–65]. Also, other studies have highlighted the role of JAK2/STAT5B in osteoblastogenesis for the implication of some transcriptional factors and proteins, such as Runx-2, BMP-7, Tbx-3 [66–69]. Nevertheless, due to the strong interaction between JAK2/STAT5B, it is not easy to associate a respective function to each molecule. As for the three members, STAT2, STAT4, and STAT6, they do not seem to be directly involved in bone remodeling. Although, studies show that STAT2 could be implicated in bone homeostasis through STAT1; while STAT4 and STAT6 are involved in inflammatory arthritis [70]. It has been shown that STAT 3 inhibition induced a reduction of RANKL levels and decrease bone resorption through a decline of osteoclast activity mediated by RANKL in several experimental animal models of inflammatory arthritis [71, 72]; accordingly, in vitro studies showed that the STAT 3 inhibition induced a reduction of RANKL-mediated osteoclast differentiation from monocytes in mice and human [73, 74]. Among all the STATs proteins, STAT1 and STAT3 play a more important role in bone maintenance. Some studies show the critical role of STAT1 in the inhibition of the osteoclastogenesis process [75, 76]. On the contrary, other studies carried out on STAT1-deficient animal models highlight the suppressor function that STAT1 has in bone formation, through its relationship with Runx2 [77].

To confirm this last hypothesis, some researchers found that the activity of osteoblasts, in the bone formation, is accelerated in the absence of STAT1 during fracture healing [78]. Further, in ovariectomized rat, STAT1/3 inhibition is associated to an increased osteoblast activity, expressed as enhanced osteocalcin al alkaline phosphatase production [79]. The key role as a transcription factor for bone cells is certainly assigned to STAT3 [33–35]. The involvement of STAT3, in addition to survival and cellular functionality, also concerns the pathways of many cytokines and growth factors [80]. A team of researchers observed swift activation and induction of STAT3 signaling in mesenchymal stem cells (MSC), important regulators of osteoblast differentiation [81]. It has been widely discussed that STAT3 partakes in bone maintenance through its expression in osteoblasts and its inactivation effectively decreases bone formation in vivo [82–84]. To confirm this, animal studies were conducted on knockout mice engineered on the CRE-loxP system, highlighting that the inactivation of STAT3 in osteoblasts leads to a lower BMD. Moreover, in a very recent study conducted by Davidson et al., it is highlighted that the participation of STAT3, during the regulation of the osteoclastogenic process, occurs in a different way in female than male osteoclastic cells. So, these data suggest that the STAT3 signal may have a key role in bone turnover [46, 84, 85]. Initially, an inactivating STAT3 mutation was found, which leads to a disease called Job syndrome. Among the characteristics found in patients with this syndrome, a reduced BMD and presence of fractures are not to be underestimated. Besides, the mutation results in increased bone resorption and a greater number of osteoclasts [86–89]. Some studies show that inhibition of STAT3 induces a decrease in the osteoclastogenesis process [52, 53]. Young et al., in their animal experiment, show that STAT3 is involved in osteoclastogenesis in vivo and it can regulate the NFATc1 factor [90].

## JAK/STAT, cytokines and bone cells

Many molecules, including cytokines, are involved in the cascade of the JAK/STAT signaling pathway. The recognition of cytokines with their receptors on the cell surface induces the activation of JAKs proteins. Consequently, the cascade activation mechanism of other components begins, such as the STATs, which, by binding to DNA, regulate the transcription processes of many genes [91]. The cytokines that are part of this pathway are more than 30, including numerous interleukins, tumor necrosis factor-α (TNF-α), interferon-α/γ (IFNα/γ), and oncostatin M (OSM), each of which is implicated in many pathologies [92]. Some cytokines (Table 1) are potentially involved in normal bone remodeling [93].

**Table 1:** Positive/Negative effect of citokines on bone cells, processes and bone remodeling

Citokine	Effect on Osteoblast and bone formation	Effect on osteoclast and bone resorption	Bibliographic references
IL-1	–	Positive effect (↑ osteoclastogenesis) (↑ bone resorption)	[37, 99, 100]
IL-3	Positive effect (↑ osteoblastogenesis) (↑ bone formation)	Negative effect (↓ osteoclastogenesis) (↓ bone resorption)	[101–105]
IL-4	–	Negative effect (↓ osteoclastogenesis) (↓ bone resorption) (↓ osteoclast activity)	[47, 106, 107]
IL-6 ●	Positive effect (↑ osteoblastogenesis) (↑ bone formation)	Negative/Positive effect ● (↓ osteoclastogenesis) (↑bone resorption) (↑ osteoclasts formation)	[94–98]
IL-7 ●	–	Negative/Positive effect ● (↓ osteoclastogenesis) (↓ bone resorption) (↓ osteoclast activity) (↑ osteoclasts formation)	[47, 106–108]
IL-12	–	Negative effect (↓ osteoclastogenesis)	[109–111]
IL-15	–	Positive effect (↑ osteoclastogenesis)	[90, 112]
IL-17	Positive effect (↑ osteoblastogenesis) (↑ bone formation)	–	[113–115]
IL-23	–	Negative effect (↓ osteoclastogenesis)	[114, 116]
IL-27	Involvement in osteoblasts (to be studied)	Negative effect (↓ osteoclastogenesis)	[114, 116]

↑, Stimulation, increase, promotion; ↓, Inhibition, decrease, induction; ●, uncertain/contrasting effect

## Interleukin-6

Interleukin-6 plays a key role in bone turnover. Recently, experimental evidence has shown that IL-6 presents two forms, *cis* and *trans*, and that IL-6 trans form promotes bone formation [94]. Other studies suggest that, with the activation of STAT3 and the involvement of receptor subunit gp130 to transduce signals, IL-6 can mightily inhibit RANKL-induced osteoclasts differentiation [95]. In contrast, IL-6 stimulates the production of RANKL in osteoblasts and it could also induce an increase in osteoclasts and bone resorption [96–98].

## Interleukin-1

Similarly, IL-1 also stimulates the process of osteoclastogenesis and bone resorption [99, 100]. A study has shown that the formation of TRAP-positive multinucleated cells (MNCs) is inhibited by using a molecule that blocks the action of IL-1 and IL-6 [37].

## Interleukin-3

IL-3 could inhibit the process of osteoclast differentiation and bone resorption. On the other hand, it stimulates the process of osteoblastogenesis and consequently an increased bone formation. Moreover, IL-3 probably modulates, through the activation of JAK2/STAT5, a greater expression of both the soluble and membrane form of RANKL in calvaria osteoblasts, despite this does not involve the maturation of multinucleated osteoclasts [101–105].

## Interleukin-4 and Interleukin-7

Some studies suggest that IL-4 and IL-7 negatively regulate the osteoclastogenesis process [106, 47–107]. Therefore IL-4, through the phosphorylation of STAT6, inhibits the osteoclasts activity and bone resorption. However, a team of researchers has found that, through STAT5 signaling, IL-7 induces the formation of osteoclasts [108].

## Interleukin-12

Several studies indicate that IL-12 also has inhibitory effects on the osteoclastogenesis process; however, the mechanism by which this inhibitory effect occurs is still being investigated. Nevertheless, it has been shown that this interleukin induces STAT phosphorylation through two of the JAKs proteins, JAK2 and TYK2, but its negative effect appears to involve both interferon g and T cells [109–111].

## Interleukin-15 and Interleuikin-17

In an animal study, in which the mice were defective of the IL-15 receptor, increased bone mass was shown, suggesting the role of IL-15 in the formation of osteoclasts [90–112]. IL-17 is of great interest in the involvement of bone and inflammatory diseases. Recent studies have indicated that Il-17 may be directly involved in the process of osteoblastogenesis and its implication in the rapid differentiation and maturation of osteoblasts results. The role of IL-17 has been investigated in bone cells of spondyloarthritis patients. Therefore, the researchers found that IL-17A induces the activity of osteoblasts through JAK2/STAT3 signaling. Furthermore, the activation of osteoblasts is drastically reduced by blocking JAK2 and IL-17A through an inhibitor [112–115].

## Interleukin-23 and Interleukin-27

Finally, IL-23 and IL-27 are also part of the gp130 family of cytokines. However, their functionality at the level of the bone compartment is still being studied. Despite this, IL-27 has been seen to induce the transcription process in osteoblasts through STAT3. Moreover, both seem to participate in the process of osteoclastogenesis by exhibiting their inhibitory effect and reducing the number of multinucleated osteoclasts [114–116].

## Conclusions

Through careful research in the literature, we wanted to focus our attention on the molecular mechanisms that affect the involvement of the JAK/STAT pathway at the cellular level, specifically at the level of bone cells, osteoblasts, and osteoclasts, and how they behave in the remodeling process bone. At the same time, the knowledge of the interaction between the immune system and the bone system has allowed us to evaluate also how cytokines, through the JAK/STAT signaling pathway, influence and participate in the mechanisms that govern bone turnover. It should be underlined that JAK/STAT inhibition showed conflicting and often opposite effects both in experimental and clinical models, in a large variety of physio-pathological relevant processes and in different tissues, including bone, probably because they can act by nonspecific mechanisms. Further, the different effects depending on cellular and tissue context and the complex interaction with other regulatory signaling pathways. The development of more specific JAK inhibitors, which target individual members of the STAT family, could contribute better to understand the role of JAK/STAT signaling in physiological and pathological conditions, including skeletal disorders. On the other hand, many studies will still be needed that allows scientists to investigate the functional mechanisms of the JAK/STAT pathway and its involvement in the bone system. This will allow us to develop new molecules and improve existing ones by inhibiting and/or blocking the JAK/STAT cascade with a view to new therapeutic strategies.
